# High-Performance Agent-Based Modeling Applied to Vocal Fold Inflammation and Repair

**DOI:** 10.3389/fphys.2018.00304

**Published:** 2018-04-12

**Authors:** Nuttiiya Seekhao, Caroline Shung, Joseph JaJa, Luc Mongeau, Nicole Y. K. Li-Jessen

**Affiliations:** ^1^Department of Electrical and Computer Engineering, University of Maryland, College Park, MD, United States; ^2^Department of Mechanical Engineering, McGill University, Montreal, QC, Canada; ^3^School of Communication Sciences and Disorders, McGill University, Montreal, QC, Canada

**Keywords:** high-performance computing, agent-based modeling, biosimulation, inflammation, wound healing, vocal fold, *in situ* visualization

## Abstract

Fast and accurate computational biology models offer the prospect of accelerating the development of personalized medicine. A tool capable of estimating treatment success can help prevent unnecessary and costly treatments and potential harmful side effects. A novel high-performance Agent-Based Model (ABM) was adopted to simulate and visualize multi-scale complex biological processes arising in vocal fold inflammation and repair. The computational scheme was designed to organize the 3D ABM sub-tasks to fully utilize the resources available on current heterogeneous platforms consisting of multi-core CPUs and many-core GPUs. Subtasks are further parallelized and convolution-based diffusion is used to enhance the performance of the ABM simulation. The scheme was implemented using a client-server protocol allowing the results of each iteration to be analyzed and visualized on the server (i.e., *in-situ*) while the simulation is running on the same server. The resulting simulation and visualization software enables users to interact with and steer the course of the simulation in real-time as needed. This high-resolution 3D ABM framework was used for a case study of surgical vocal fold injury and repair. The new framework is capable of completing the simulation, visualization and remote result delivery in under 7 s per iteration, where each iteration of the simulation represents 30 min in the real world. The case study model was simulated at the physiological scale of a human vocal fold. This simulation tracks 17 million biological cells as well as a total of 1.7 billion signaling chemical and structural protein data points. The visualization component processes and renders all simulated biological cells and 154 million signaling chemical data points. The proposed high-performance 3D ABM was verified through comparisons with empirical vocal fold data. Representative trends of biomarker predictions in surgically injured vocal folds were observed.

## 1. Introduction

### 1.1. Agent-based modeling (ABM)

Agent-based modeling is a widely used approach to quantitatively simulate dynamical systems (Macal, 2016). The popularity of ABMs can be observed in the variety of ABM frameworks developed in the past decade (for reviews, please see An et al., 2009; Gorochowski, 2016; Hellweger et al., 2016; Macal, 2016). Each ABM is defined by a set of autonomous *agent*s whose interactions among themselves and with their environment are governed by a number of stochastic or deterministic rules (Hellweger et al., 2016; Macal, 2016). In contrast to equation-based approaches, ABMs are decentralized. That is, the system's behavior is determined by the collective behavior of each individual *agent* in the system. Although a universal definition of ABMs remains debatable (Macal, 2016), fundamental components of ABM typically include: agent set, agent relationship set, and agents' environment (Macal and North, 2010).

Firstly, a set of agents includes the agents themselves, their attributes and their behavioral rules. Agents' behavioral rules govern their decisions and actions. In ABM, *agent*s can represent a wide spectrum of individual entities such as consumers, markets, and geographic regions in economic models (Tesfatsion, 2006; Caiani et al., 2016), animals in ecosystems (McLane et al., 2011, 2017), and biological cells and proteins in systems biology models (D'Souza et al., 2009; Krekhov et al., 2015; Shi et al., 2016). Secondly, the set of “agent relationships and methods of interactions” (Macal and North, 2010) defines the criteria of a group of entities each agent is bound to interact with, and how these interactions are carried out. For instance, some ABMs may allow agents to interact only *directly* with other agents, some may allow only *indirect* interactions while some may allow both (Ausloos et al., 2015). A *direct* interaction represents an immediate impact one agent leaves on another. Particle collision is an example of a direct interaction, where colliding particle agents affect the states of each other directly. On the other hand, *indirect* interactions have been used to mimic the lingering effects of transmitted signals (Godfrey et al., 2009; Crandall et al., 2010; Richardson and Gorochowski, 2015; Gorochowski and Richardson, 2017). An example of indirect agent interaction includes chemical secretion as a form of inter-cellular communication. This chemical secretion example is classified as indirect because the agents alter the states of the environment to communicate, rather than altering the states of the recipient agents directly. Lastly, the agents' environment houses the autonomous agents. This space can be discrete lattice-based (Wilensky and Evanston, 1999), continuous lattice-free (Van Liedekerke et al., 2018), or hybrid (Chooramun et al., 2012). The environment may maintain local attributes depending on the application and underlying implementation (Drasdo et al., 2018).

Our first published ABM (Li et al., 2008) was programmed on the platform of Netlogo and thus most of the terminology used herein was adopted from the dictionary of NetLogo (Wilensky, 2015). In our implementation, the 3D environment, also known as the ABM *world*, represents a human tissue. The 3D environment is spatially discretized into rectangular volumes called 3D *patch*es. Each mobile *agent* represents an inflammatory cell that can move from one patch to an adjacent patch and make decisions to perform certain actions at discrete time steps. *Agent*s make decisions based on the state of the *patch*es, which allow them to alter their environment to interact indirectly with other *agent*s. Chemokines and extracellular matrix (ECM) proteins are associated with the states of the patches.

### 1.2. Computational challenges

The simulation of high-resolution ABMs in biology (Bio-ABM) often deals with large data sets. Processing a large amount of data demand significant computational resources. To address the challenges of the significant computational demands of large-scale ABMs, multiple high-performance computing (HPC) ABM tools have been developed over the years. These tools have also been used to parallelize bio-ABMs. For example, FLAME (Kiran et al., 2010; Coakley et al., 2012) is an implementation of an ABM framework for parallel architectures based on stream X-machines. FLAME has been used to speed up the simulation of ecological systems in various fields including systems biology (Richmond et al., 2010). FLAME GPU (Richmond et al., 2009; Richmond and Chimeh, 2017) and SugarScape on steroid (D'Souza et al., 2007) represent efforts to support ABM acceleration on GPU platforms. These tools have demonstrated their applicability to biological system simulations such as tissue wound and disease modeling (D'Souza et al., 2009; Richmond et al., 2010; de Paiva Oliveira and Richmond, 2016). Repast HPC (Collier and North, 2013) was developed as an MPI extension to its predecessors, Rapast and Repast Symphony (Collier, 2003; North et al., 2005). Repast HPC was adopted to accelerate the simulation of bone tissue growth (Murphy et al., 2016).

Multiple HPC ABM tools have also been developed specifically for systems biology applications. An example includes a Repast-based framework for single-cells and bacterial population called AgentCell (Emonet et al., 2005). The AgentCell framework provides support for running multiple non-interacting single-cell instances concurrently on massively parallel computers. More examples include HPC ABM frameworks for multi-core CPUs such as CompuCell3D (Swat et al., 2012a,b), CellSys (Hoehme and Drasdo, 2010), and Morpheus (Starruß et al., 2014). These frameworks target multi-core CPU acceleration on a single compute node using OpenMP. In addition, other techniques have been proposed to accelerate specific biological models on multi-core CPUs or GPUs (Christley et al., 2010; Falk et al., 2011; Zhang et al., 2011; Cytowski and Szymanska, 2014). However, none of the aforementioned HPC ABM techniques or tools exploit the computing power of both CPUs and GPUs simultaneously, resulting in a sub-optimal resource utilization.

Another significant challenge in systems biology modeling lies in the multi-scale nature of the model (Dallon, 2010; Eissing et al., 2011; Cilfone et al., 2014; Schleicher et al., 2017). To ensure optimal performance, it is important for differences in spatiotemporal scales between cellular and chemical interactions to be handled in a cost-effective manner. Cellular movements occur at a rate of micrometers per hour (μ*m*/*h*), while cytokine diffusion in tissue occurs at a rate of micrometers per second (μ*m*/*s*). A naive approach would be to iteratively simulate the model at the smallest temporal scale required. However, this approach would result in a prohibitive increase in the computational cost. A possible solution is to use coarse-graining techniques to lower the computational intensity (Qu et al., 2011). The concept of coarse-graining in ABM refers to the simulation of *super-agents* whose rules represent aggregated behaviors of smaller units (Chang and Harrington, 2006; Maus et al., 2011; Sneddon et al., 2011). Our earlier 2D framework uses a mechanism that captures the behavior of multiple iterations of the finer-scale processes, i.e., chemical diffusion, over a coarse time window using convolution (Seekhao et al., 2016). This intensive computation is then offloaded to a single GPU while the CPU cores focus on coarse-grain cellular processes.

An effective visualization component is essential for understanding the progress of the simulation and emerging trends. However, with billions of data points being produced after each iteration, implementing real-time visualization is not trivial. Usually, visualization is performed on pre-simulated/pre-processed data that are stored on disk. Such a method is known as *post-hoc* visualization. On the other hand, large simulation data sets have prompted work on coordinating the simulation and visualization simultaneously, also known as *in situ* visualization (Rivi et al., 2012; Nvidia, 2014). *In situ* visualization allows the outputs to be analyzed on the same machine that produced them. The ability to perform on-site data analysis reduces the amount of data movements between the server and remote users. This property makes *in situ* visualization an ideal way to visualize simulations that produce large data sets such as our case. Paraview Catalyst (Bauer et al., 2013; Ayachit et al., 2015) and work reported in Kuhlen et al. (2011) are examples of libraries developed to enable *in situ* processing of simulation output on popular existing visualization frameworks such as Paraview (Henderson et al., 2004) and VisIt (Childs et al., 2005). A bitmap-based and a quadtree-based ABM approach (Krekhov et al., 2015; Su et al., 2015) were proposed respectively to analyze the numerical output *in situ* and reduce non-essential simulation data. Most of these strategies were able to reduce the disk loads, but still required disk storage for the remaining essential data. In the present work, similar to (Seekhao et al., 2016, 2017), VirtualGL was employed as a tool for developing *in situ* visualization of an ABM that circumvents disk storage and directly visualize simulated outputs written on to a RAM. This real-time visualization feature would assist researchers in tracking the progress and steering the course of the simulation.

### 1.3. Case study—vocal fold inflammation and repair

#### 1.3.1. Problem background

In the United States, voice problems were estimated to affect one in 13 adults annually (Bhattacharyya, 2014). In one study, nearly one third of the sampled population has experienced voice disorder symptoms at some point in their lifetime (Roy et al., 2004). In particular, voice disorders constitute a major occupational hazard in many professions such as salespeople, teachers, performing artists, attorneys, and sport coaches, due to the intensive vocal demand of the job (Vilkman, 2000; Verdolini and Ramig, 2001; Jones et al., 2002; Fellman and Simberg, 2017). The estimated lifetime prevalence of voice disorders is as much as 80% in occupational voice users (Cutiva et al., 2013; Martins et al., 2015). Human vocal folds are under continuous biomechanical stress during voice production. Excessive phonatory stress can induce a cell-mediated inflammatory response and structural tissue damage, leading to a pathological condition (Gunter, 2004; Li et al., 2013; Kojima et al., 2014). Patients with phonotraumatic lesions are usually prescribed behavioral voice therapy (Johns, 2003; Misono et al., 2016) or surgical excision of the lesion in combination with various adjunctive treatments (Hansen and Thibeault, 2006; Hirano et al., 2013; Ingle et al., 2014; Moore et al., 2016). Unfortunately, the healing outcome of voice treatments often depend on the lesion, the treatment dose, and the patient's vocal needs (Abbott et al., 2012; Roy, 2012; Li N.Y. et al., 2014). The success rate of voice treatment varies extensively between 30 and 100% (MacKenzie et al., 2001; Zeitels et al., 2002; Wang et al., 2014; Vasconcelos et al., 2015), making the treatment planning process difficult for voice therapists and surgeons. The unpredictable treatment outcome is axiomatic and takes a huge toll on a person's career, a clinician's decision-making process and society's healthcare costs. A computational tool that can estimate voice treatment success would spare patients from unnecessary and costly treatments and potentially harmful side effects.

Computer simulations have become central to personalized medicine (Deisboeck, 2009; Chen and Snyder, 2012; Li et al., 2016; Canadian Institutes of Health, 2017). This approach involves the creation of computational models to estimate treatment outcome and identify the best possible treatment for a given patient. Simulation modeling involves the integration of the best available knowledge into a computer platform to represent the real-world problem. The process involves an abstraction of causal relationships between patient variables and health outcomes followed by a rigorous and iterative protocol of model calibration and validation (Galea et al., 2009; Marshall and Galea, 2014; O'Donnell et al., 2016). The property that sets numerical simulation models apart from standard statistical models is the observability of the evolution of patient behaviors and health conditions in the computer model as time passes during simulation. Such an approach provides a computational tool for clinicians to evaluate the impact of intervention or other modifiable variables on health outcomes in advance or along any point during the intervention.

Computer models have been developed for complex health conditions, including sepsis (Clermont et al., 2004; Kumar et al., 2004; Vodovotz et al., 2006), traumatic brain injury (Vodovotz et al., 2010), acute liver failure (Wlodzimirow et al., 2012), diabetes (Boyle et al., 2010; Day et al., 2013), obesity (El-Sayed et al., 2013; Hammond and Ornstein, 2014), and cardiovascular disease (Hirsch et al., 2010; Li Y. et al., 2014; Li et al., 2015). In our case, a series of ABMs have been developed to numerically simulate the essential biology underlying vocal injury and repair with the goal of helping clinicians to better tailor treatments for patients with voice disorders (Li et al., 2008, 2010a,b, 2011; Miri et al., 2015; Seekhao et al., 2016).

In the current study, an existing high-performance 2D ABM (Seekhao et al., 2016) is substantially enhanced to a much larger 3D model in an attempt to faithfully capture the physiological dimension of human vocal folds. A diffusion kernel reduction technique is used to enhance the performance and ensure that all necessary 3D data required for diffusion fits within the GPU global memory. A scheduling scheme for a heterogeneous compute node, which consists of multi-core CPU and many-core GPUs, is then used to completely mask the execution time of the computationally intensive diffusion and visualization tasks. This low-cost, high-resolution, and high-performance computing ABM platform with real-time visualization capability is an original concept in disease modeling, and can make complex disease models practical in clinical settings.

#### 1.3.2. Modeling vocal fold repair with ABM (VF-ABM)

**Figure 1:**
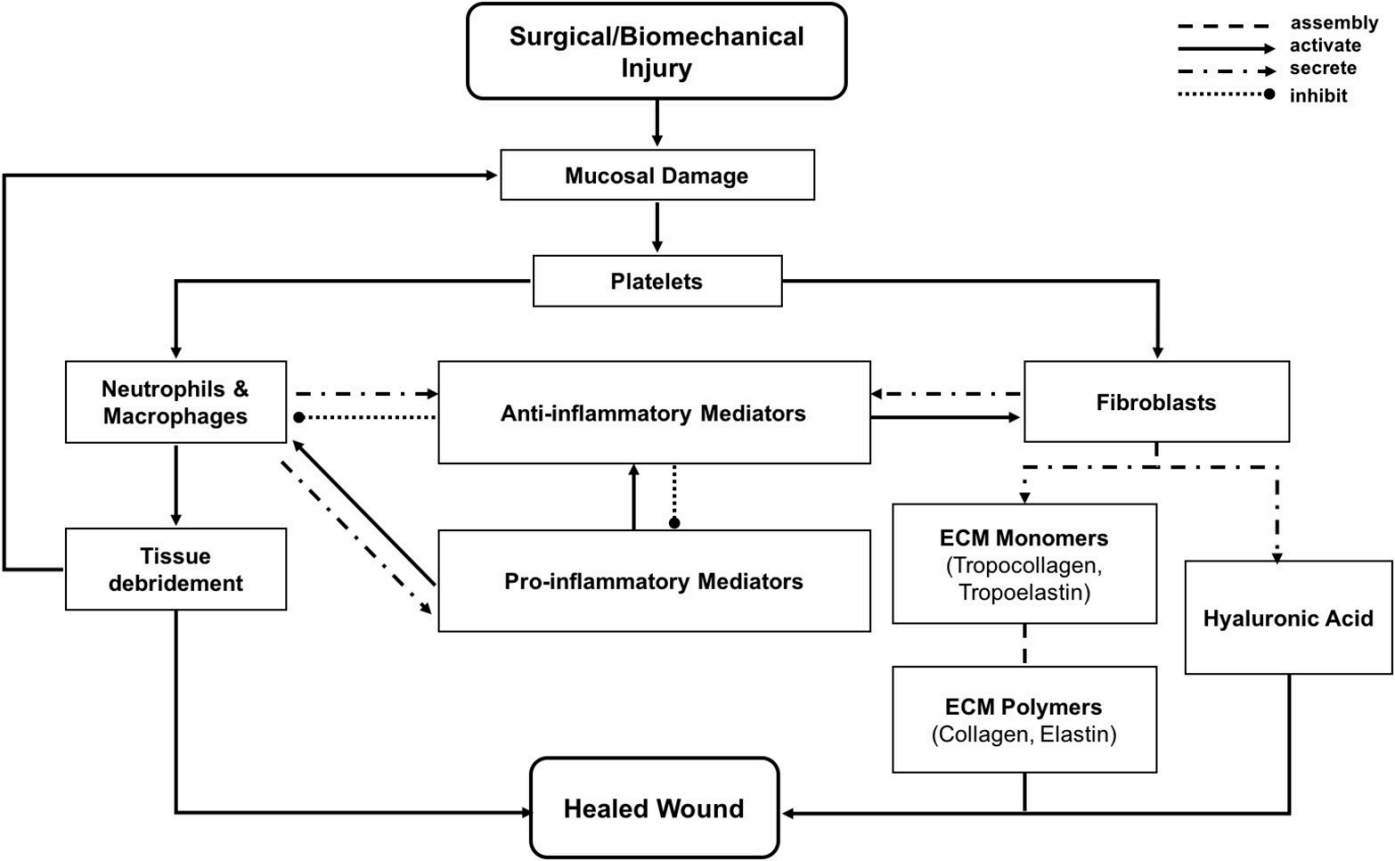
Flowchart of vocal fold inflammation and healing events in the ABM. This diagram is modified from Li et al. (2008).

In the vocal fold ABM (VF-ABM) used in this work, the inflammatory cells were implemented as *agents* (Li et al., 2008, 2010b, 2011). The chemokines and ECM proteins were implemented as states of the *patches*. The aggregation of these components yields the state of the vocal fold (ABM *world*) at each given point in simulated time. Table 1 summarizes the roles that each type of cell agent plays in the healing process. At the time of acute injury, the traumatized mucosal tissue within the damaged area triggers platelet degranulation. Different chemokines get secreted resulting in vasodilation stimulation and attraction of inflammatory cells, namely, neutrophils and macrophages to the wound site. Activated neutrophils and macrophages at the wound area further secrete chemokines to attract fibroblasts and remove cell debris. To repair the wound, activated fibroblasts proliferate and deposit ECM proteins such as collagen, elastin, and hyaluronan. These ECM proteins then form a scaffold for supporting fibroblasts in wound contraction, cell migration, and other wound repair activities (Bainbridge, 2013). The flow diagram of the interactions between all the components in the model is shown in Figure 1 (modified from Li et al., 2008). In each iteration, the VF-ABM executes the following major steps:

- **Seed Cells**—Cell recruitment from surrounding native tissues to the damaged area.
- **Cell Function**—Cell migration, proliferation, cytokine production and ECM production (Table 1).
- **ECM Function**—Tissue repair. Fragments of ECM protein acting as danger signals.
- **ECM Fragmentation**—Fragmentation of ECM proteins if TNF-α or MMP is beyond a threshold.
- **Chemical Diffusion**—Mass diffusion of each of the chemical signals including TNF-α, TGF-β, FGF, MMP8, IL-1β, IL-6, IL-8, and IL-10.

**Table 1:** Summary of agent rules.

**Agent**	**Actions**
Platelets	Secrete TGF-β1 and IL-1β to attract other cells and regulate ECM protein production.
	Secret MMP8 to promote collagen fragmentation.
Neutrophils	Secrete TNF-α to attract and promote activation of other neutrophils and macrophages. TNF-α also plays a role in regulating the production and fragmentation of ECM proteins.
	Secrete MMP8 to promote collagen fragmentation.
Macrophages	Secrete TNF-α, TGF-β1, FGF, IL-1β, IL-6, IL-8, IL-10 to attract cells, regulate cell activation, fibroblast proliferation, ECM protein production, and ECM protein fragmentation.
	Clean up cell debris.
Fibroblasts	Secrete TNF-α, TGF-β1, FGF, IL-6, IL-8 to attract cells, promote cell activation and regulate fibroblast activation, and promote ECM fragmentation and regulate ECM production.
	Secrete ECM proteins to repair tissue damage.
ECM Managers	Manages ECM functions and conversion. One manager per patch.

## 2. Materials and methods

The 3D ABM simulation suite includes both computation and visualization components. The computational tasks can be categorized as coarse- or fine-grain. Coarse grain tasks include inflammatory cell and ECM functions, which involve more complex control structures and relatively small data movements. On the other hand, fine-grain tasks include the diffusion of the different chemicals, which involves relatively simple operations applied to large amounts of data. In this section, we start by describing our hardware and software environment. We will then discuss how task assignments and coordination are performed to ensure correct synchronization and maximize load balance. Finally, we will describe how each task category underwent optimization specific to its computational and data access characteristics. The model size and configuration details are summarized in **Table 3**. The source code of the VF-ABM prototype with optimizations described in this work can be found at https://github.com/VF-ABM/hpc-abm-vf-version_0_6.

### 2.1. Hardware and software environment

Our high-performance VF-ABM was tested and benchmarked on a compute node with a 44-core Intel(R) Xeon(R) CPU E5-2699 v4 @ 2.20GHz host and two attached accelerators, NVIDIA Tesla M40. Table 2 summarizes the GPU specifications. Each Tesla M40 GPU consisted of 3,072 cores per device with 24 GB of global memory. C++, a lightweight programming language, was used to implement the program to ensure fast and efficient simulation. To utilize the multiple CPU cores available, Open Multi-Processing (OpenMP) was used to parallelize coarse-grain cellular processes. OpenMP is a highly portable Application Programming Interface (API) that supports multi-threading on shared-memory platforms via a set of platform-independent compiler directives (Dagum and Enon, 1998). OpenMP was further used to allocate separate threads to communicate and launch tasks on the GPUs. Chemical diffusion tasks were offloaded to the GPUs due to their high computational needs. These tasks were programmed using the NVIDIA Compute Unified Device Architecture (CUDA) (Nvidia, 2007) model. CUDA is a parallel computing platform and programming model, which allows general purpose multi-threaded programming of GPUs via C-like language extension keywords. In the CUDA language, a GPU is presumed to be attached to the host (CPU), which controls data movement to/from the GPU. The CPU is responsible for launching kernels, which are functions to be executed by all threads launched on the GPU. Open Graphics Library (OpenGL) was used to implement the visualization component of the simulation. OpenGL is an open standard, cross-language API for 2D and 3D rendering. OpenGL is widely used over a broad range of graphics applications due to its portability and speed.

**Table 2:** Summary of NVIDIA Tesla M40 GPU specifications.

**GPU**	**Tesla M40**
SMs (per Device)	24
CUDA Cores per SM	128
Registers per SM	64k
L2 cache size	3.0 MB
Global memory (per device)	22.4 GB
Max clock rate	1.11 GHz
Memory clock rate	3.0 GHz
Memory bandwidth	288 GB/s
Compute capability	5.2

### 2.2. Scheduling and coordination of CPU-GPU computation and visualization

The 3D VF-ABM consisted of an environment with 154 million patches (Table 3). Each patch stored information of ECM proteins and chemical data. In addition, around 17 million mobile agents, representing the inflammatory cells, resided in this ABM world. The model simulated the dynamic biological processes pertinent to vocal fold inflammation and repair at 30 min time intervals. At each model iteration, the operations corresponding to ECM functions, chemical diffusion, and cell (agent) functions were executed followed by the update of the ABM world. Given the computational complexity and the amount of data involved, each iteration required a careful mapping and scheduling of these operations on the available hardware resources. In addition, the visualization provides essential spatial information of ECM proteins, chemicals, and inflammatory cells during the simulation. The overall goal was to simulate and visualize the 3D VF-ABM as fast as possible for each iteration.

**Table 3:** Summary of human simulation configurations.

**Item**	**Unit**	**Size**
World		
Size	Patches × patches × patches	1,390 × 1,006 × 110
	mm × mm × mm	20.85 × 15.09 × 1.65
Patch size	μm × μm × μm	15 × 15 × 15
Total number of patches	Unit	154 million
ECM data	Types	3
	Data points	461 million
Chemical data	Types	8
	Data points	1.2 billion
Inflammatory cells (initial)		
Neutrophils	Cells	1.72 million
Macrophages	Cells	0.97 million
Fibroblasts	Cells	12.20 million
Simulated time-step	Minutes	30

The typical approach to tackle such computational complexity has been to use multi-core CPUs and many-core GPUs. Accelerators such as GPUs need a CPU host, and each of the GPU and CPU has a number of cores that can be exploited using parallel programming techniques. However, GPUs have received much more attention in general whenever accelerated performance is the main goal due to their extremely high performance in data parallel computations. Often, this hardware preference results in idle CPUs, waiting for GPUs to perform all the work after the dispatch of the computing tasks to the GPUs. In this work, the aim was to exploit the resources available on both the CPU and GPU simultaneously so as to achieve the best possible performance. In fact, a host-device computation overlap technique was used in our earlier work, resulting in much improved performance for the 2D ABM framework (Seekhao et al., 2016). However, the 3D ABM framework was substantially more computationally demanding. The previous methods were thus further developed to achieve the desired high speed simulation and visualization necessary for the 3D ABM framework.

To achieve optimal resource utilization, it is important to address the challenges of load balancing, minimizing data movements between the CPU and GPU, and coordinating the tasks on various devices. As we moved from 2D (Seekhao et al., 2016) to 3D, the computational complexity of the simulation and the amount of data involved increased substantially. Furthermore, the execution time of the visualization component, which was negligible in the 2D simulation, became significant. Therefore, the issues of task assignment, load balancing, and device coordination need to be revisited and addressed properly.

**Figure 2:**
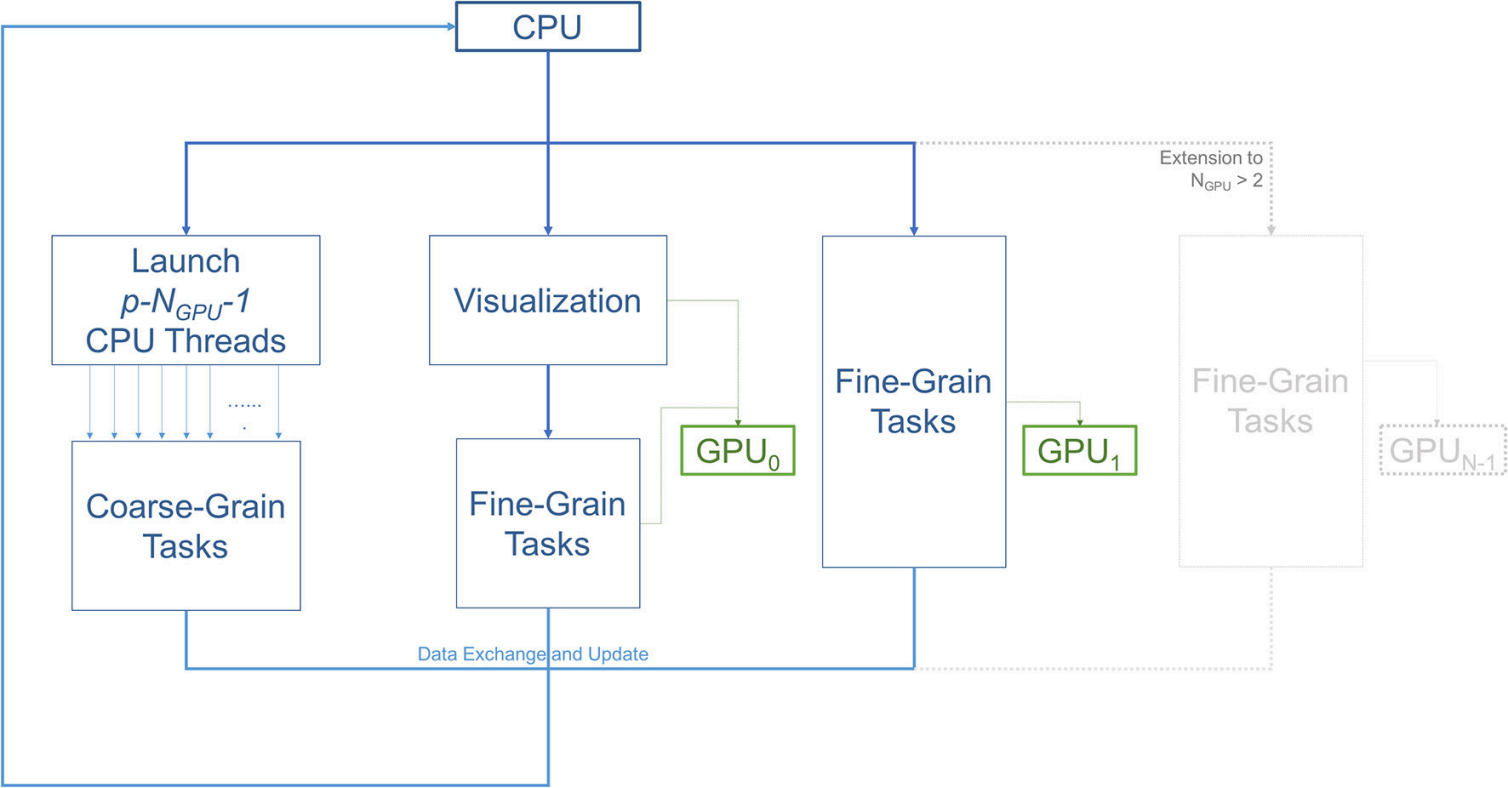
Diagram illustrating the workflow of the three main types of tasks; coarse-grain (CPU), fine-grain (GPU), and visualization. The number of GPUs is two in this setup. However, this scheme can be extended to use more GPUs as demonstrated in the gray part of the diagram. One of the GPUs is used for diffusion (fine-grain tasks), while the other is used for both visualization and diffusion. With *p* available CPU cores, *p*−*N*_*GPU*_−1 or *p*−3 threads are allocated for coarse-grain functions. The other *N*_*GPU*_ threads are in charge of managing data transfers and dispatching fine-grain tasks to the GPUs, and the last thread is spared for visualization.

Figure 2 illustrates the workflow of the 3D ABM simulation during each iteration. Specifically, it describes the task allocation on a platform consisting of a single multicore CPU with *N*_*GPU*_ GPUs attached to it. For our specific setup consisting of 2 GPUs, the simulation started on the CPU host, and then split into three paths: coarse-grain, fine-grain/visualization, and fine-grain. Each of the paths was run on separate hardware resources. The first path spawned multiple CPU threads to execute coarse-grain tasks on CPU cores. The second path was responsible for visualization and some of the fine-grain tasks that execute on a single GPU resource. The remaining fine grain tasks executed on the rest of the GPUs. All paths met at the end to exchange and update the ABM world.

The overlap of visualization and computational components required a careful device coordination as these components now shared computing resources. Algorithm 1 describes, at a high-level, how to map tasks and perform host-devices synchronization. Each GPU task, computational or visualization, has its own CPU thread for data management and communication with the GPUs. Nested CPU threads were launched at three levels. At the first level, the driver started the execution by initializing the simulation and launching two threads, one for visualization and the other for computation. The visualization rendered the current state of the ABM world using an available GPU, and then broadcast the completion of the rendering task. Concurrently with the visualization execution, the computation started by launching two more threads at the second level. Both threads at this level further launched multiple threads at the third level, depending on the number of cores available. More specifically, the first thread at level 2 was responsible for executing CPU tasks, which launched parallel threads for coarse-grain task parallelization i.e., level 3. The second thread at level 2 spawned *N*_*GPU*_ level-2 threads to launch fine-grain computation tasks on available GPUs. Note that if the visualization was not yet completed, one of the GPUs would not be available and the fine-grain tasks will have to wait (Algorithm 2). If a fine-grain task had grabbed the same GPU used for visualization, it would have to broadcast its completion so that the visualization can proceed.

**Algorithm 1:**
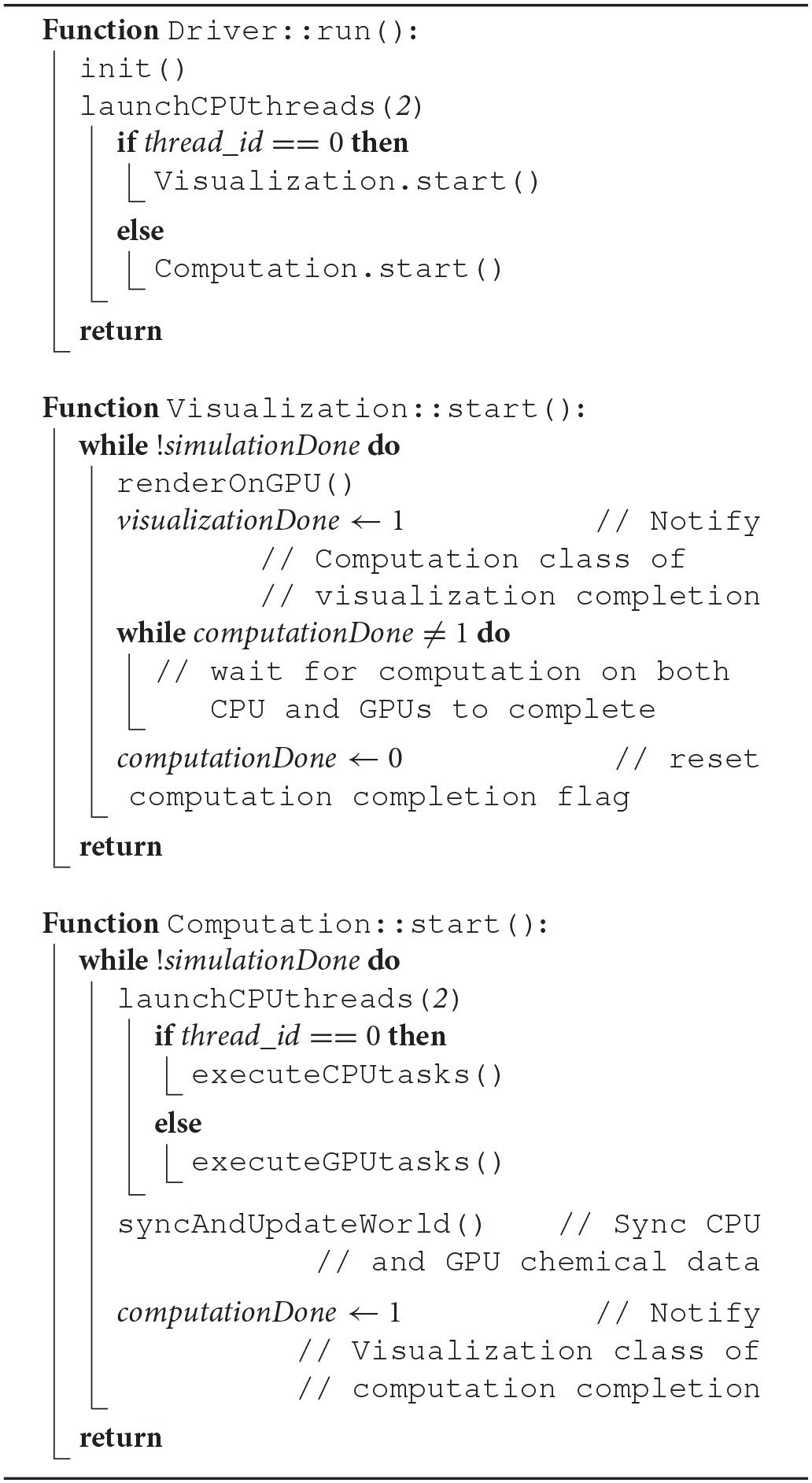
Pseudocode describing CPU-GPU scheduling related functions in Driver, Computation and Visualization class

**Algorithm 2:**
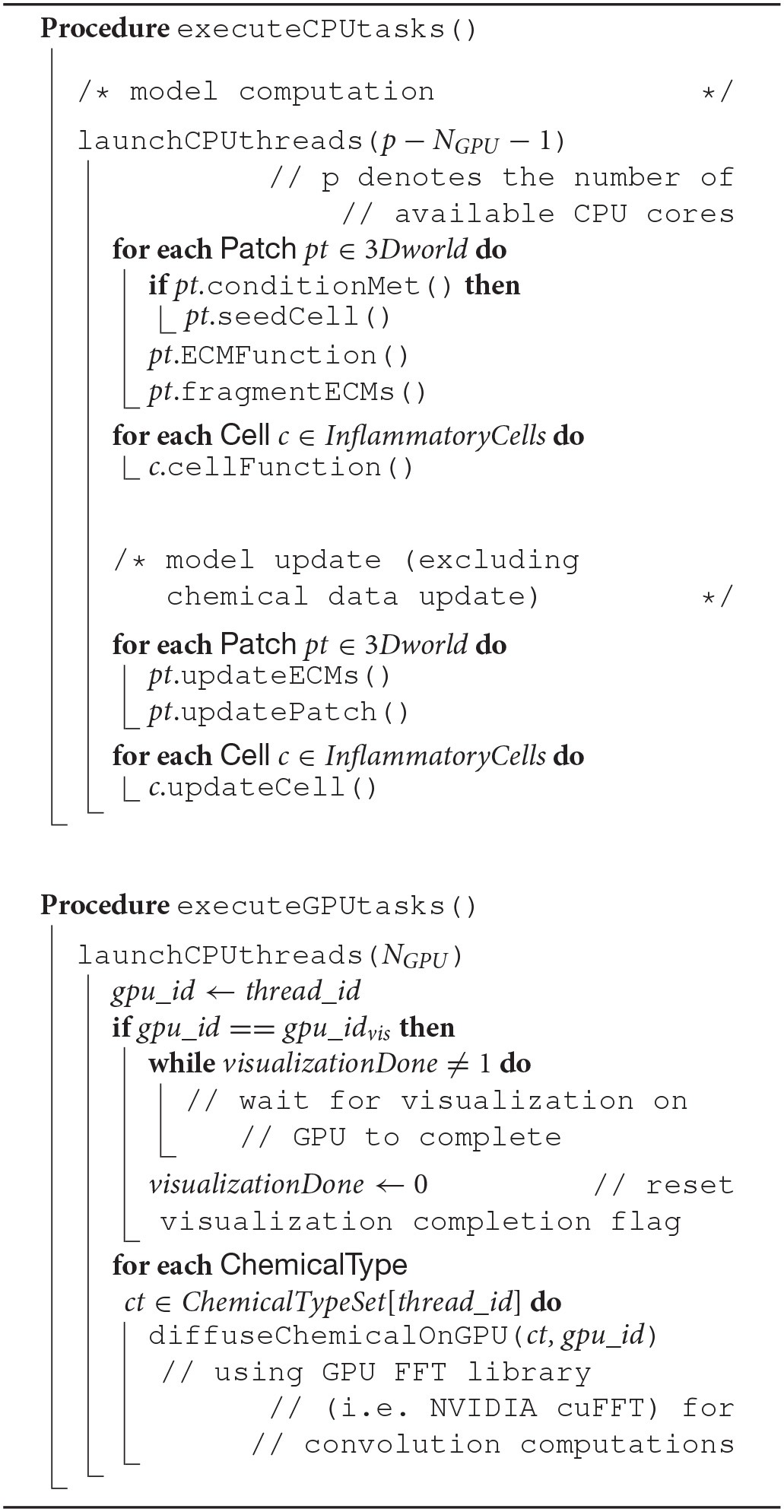
Pseudocode describing VF-ABM operations and workflow

### 2.3. Computational optimization of diffusion

Chemical diffusion was the most demanding computational component of the model. As previously mentioned, its computational demand was primarily a result of the extremely small spatiotemporal scale and high rate at which chemical diffusion occurs. To reduce the computational load, a convolution-based method was used to simulate the diffusion process (Seekhao et al., 2016). A Fast Fourier transform (FFT) was then used to reduce the complexity of convolution computations. Lastly, kernel size reduction was achieved by extracting the most dense segment of the Gaussian kernel to optimize the diffusion performance. Note that, since we deal with regular grids for the ABM world, finite difference method (FDM) is used as opposed to the more computationally intensive integral schemes.

#### 2.3.1. FFT-convolution-based diffusion

In 3D, the diffusion equation with decay can be written as

(1) $$\frac{\partial c}{\partial t}=D\left(\frac{\partial^{2}c}{\partial x^{2}}+\frac{\partial^{2}c}{\partial y^{2}}+\frac{\partial^{2}c}{\partial z^{2}}\right)-\gamma c,$$

where *c* is the chemical concentration, *D* is the diffusion coefficient and γ is the decay constant. Assuming that Δ*x* = Δ*y* = Δ*z*, and using a Taylor expansion to discretize the continuous 3D diffusion equation, we get

(2) $$\begin{array}{l} c\left(x,y,z,t+\Delta t\right)=\left(1-\frac{4D\Delta t}{\Delta x^{2}}-\gamma \Delta t\right)c\left(x,y,z,t\right)+\\ \;\frac{D\Delta t}{\Delta x^{2}}[c\left(x+\Delta x,y,z,t\right)+c\left(x-\Delta x,y,z,t\right)+\\ \;c\left(x,y+\Delta y,z,t\right)+c\left(x,y-\Delta y,z,t\right)+\\ \;c\left(x,y,z+\Delta z,t\right)+c\left(x,y,z-\Delta z,t\right)] \end{array}$$

subject to the stability constraints

(3) $$\Delta t\leq \frac{\Delta x^{2}}{6D}.$$

As shown in Table 4, the largest value of *D* in the set of chemical types in VF-ABM is $900\;\frac{\mu m^{2}}{min}$ (Spiros, 2000), with patch width Δ*x* = 15μ*m*. The condition Δ*t* ≤ 2.5 s needs to hold to meet stability constraints. Clearly, the complexity of the simulation would be unnecessarily high if the model evolved at Δτ = 2.5 s rather than Δτ = 30 min or 1, 800 s.

**Table 4:** Effective diffusion coefficients used in 3D VF-ABM.

**Effective Diffusitivity** (***μm***^**2**^/***minute***)							
**TNF-**α	**TGF-**β**1**	**FGF**	**MMP8**	**IL-1**β	**IL-6**	**IL-8**	**IL-10**
900	780	780	780	900	810	900	900

*TNF-α, TGF-β1, IL-1β, and IL-6 values are taken from Spiros (2000)*.

By letting $\lambda =\frac{D\Delta t}{\Delta x^{2}}$, Equation (2) can be rewritten as

(4) $$\begin{array}{l} c\left(x,y,z,t+\Delta t\right)=\left(1-6\lambda -\gamma \Delta t\right)\cdot c\left(x,y,z,t\right)\\ \;\lambda \cdot c\left(x+\Delta x,y,z,t\right)+\lambda \cdot c\left(x-\Delta x,y,z,t\right)+\\ \;\lambda \cdot c\left(x,y+\Delta y,z,t\right)+\lambda \cdot c\left(x,y-\Delta y,z,t\right)+\\ \;\lambda \cdot c\left(x,y,z+\Delta z,t\right)+\lambda \cdot c\left(x,y,z-\Delta z,t\right) \end{array}$$

or,

(5) $$\begin{array}{l} c\left(x,y,z,t+\Delta t\right)=\\ \;\sum_{i=x-1}^{x+1}\sum_{j=y-1}^{y+1}\sum_{k=z-1}^{z+1}c\left(i,j,k,t\right)\cdot f\left(x-i,y-j,z-k\right), \end{array}$$

where

$$f\left(x,y,z\right)=\{\begin{array}{l} 1-6\lambda -\gamma \Delta t\;x=0\wedge y=0\wedge z=0\\ \lambda \;x=\pm 1\wedge y=0,\wedge z=0,or\;\\ \;y=\pm 1\wedge x=0,\wedge z=0,or\;\\ \;z=\pm 1\wedge x=0,\wedge y=0\;\\ 0\;otherwise.\; \end{array}$$

Clearly, Equation (2) is equivalent to Equation (11), thus *c*(*x, y, z, t* + Δ*t*) = *c*(*x, y, z, t*) * *f*(*x*), where * represents the convolution operation. To compute *c*(*x, y, z*, τ + Δτ), where Δτ = *m* · Δ*t*, the chemical concentrations from the previous step, *c*(*x, y, z*, τ), is convolved with *f*(*x, y, z*), *m* times. The commutative property of convolution implies that convolving *f*(*x, y, z*) with itself *m* times results in *f*_*m*_(*x, y, z*), and the diffused concentrations at each iteration can be computed as

(6) $$c\left(x,y,z,\tau +\Delta \tau \right)=c\left(x,y,z,\tau \right)*f_{m}\left(x,y,z\right).$$

The diffusion computation can thus be accelerated by computing Equation (13) at a large time step, Δτ, without violating stability constraints. The effective diffusitivity of IL-1β in tissue, for example, is 900 $\frac{\mu m^{2}}{min}$ (Spiros, 2000). In a 15 μ*m* patch world, a 30-min time step implies that the program has to calculate *c*(*x, y, z*, τ) * *f*_720_(*x, y, z*) at each time step. In other words, a chemical on a given patch (*x*,*y*,*z*) has a spatial diffusion range of *x* ± 720, *y* ± 720 and *z* ± 720, within a window of dimension 1, 441 × 1, 441 × 1, 441, which covers approximately 3 billion patches.

#### 2.3.2. Kernel reduction

The diffusion kernel was computed by convolving the initial coefficient function, *f*(*x, y, z*), in Equation (11), with itself *m* = Δτ/Δ*t* times, where Δτ is the biological time step of 30 min and Δ*t* = Δ*x*^2^/6*D* is the diffusion time step subjected to the stability constraints (Equation 6). As calculated earlier, the effective diffusitivity of IL-1β of 900 $\frac{\mu m^{2}}{min}$ results in a 1, 441 × 1, 441 × 1, 441 kernel.

**Figure 3:**
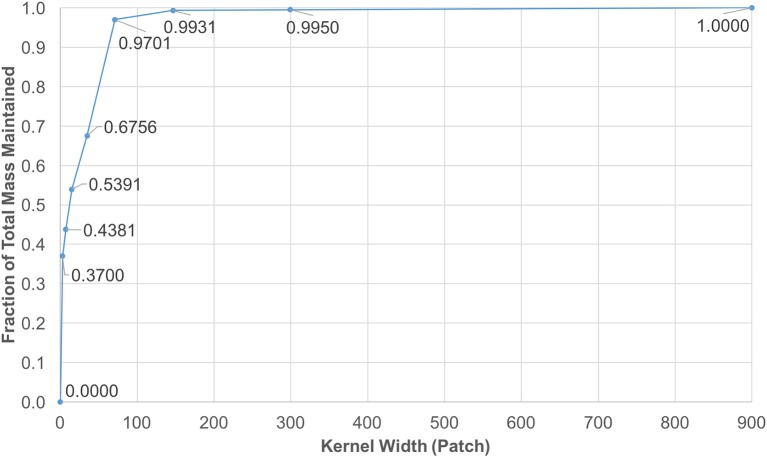
Diffusion kernel reduction mass vs. kernel width. This plot shows mass coverage with respect to extracted window width. The size of each kernel is *width*^3^ patches. It is observed that by cutting down the size from 1, 441^3^ down to 147^3^, only a fraction of 0.0069 of the mass is lost in each iteration.

Note that *f*(*x, y, z*) is smoother as it gets convolved with itself, thus a Gaussian shaped diffusion kernel is obtained. The values in Gaussian distributions are highest at the center. These values decrease and approach *zero*, the further they are from the center. This observation enabled the reduction of the kernel size by focusing on the center window, while keeping almost 100% of kernel mass. The coverage levels of the kernel mass with respect to extracted window sizes are plotted in Figure 3.

### 2.4. Visualization optimization

The 3D VF-ABM processes at least 17 million agents in each iteration while producing 1.23 and 0.46 billion chemical data and ECM protein data points, respectively. The model currently does not visualize the state of the ECM proteins on each individual patch, but rather outputs the aggregated ECM protein statistics at the end of the simulation. Due to the screen space, the user can only select one out of eight types of chemicals to be visualized in each frame. The visualization component is thus responsible for visualizing 17 million biological cells and 154 million chemical data points. To optimize the visualization of such a large amount of data, sampling was used and its effects on output simulation and corresponding performance enhancements were studied. The performance evaluation is reported in section 3.1.2.

**Figure 4:**
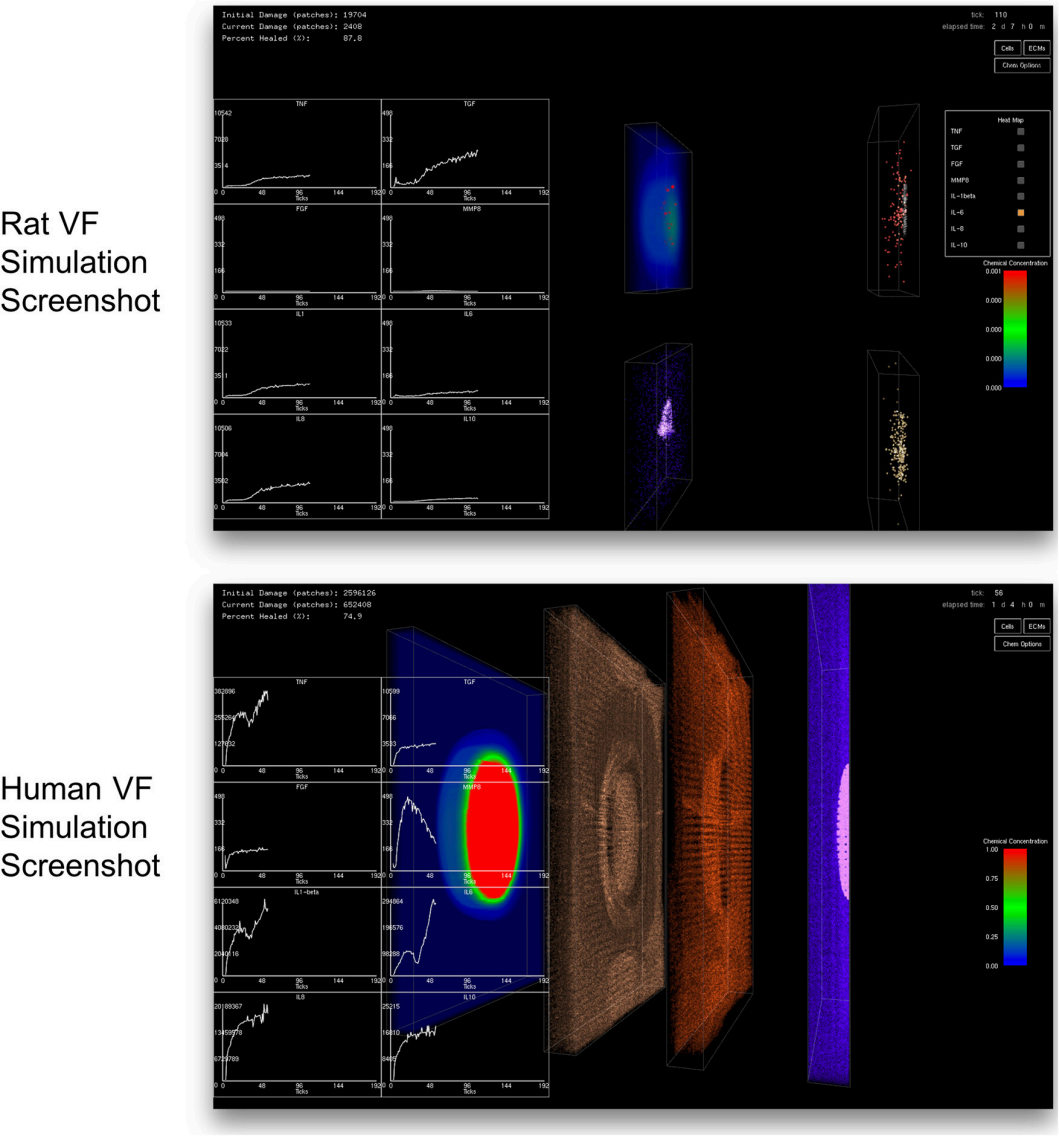
Screenshot of simulation suite captured at the client side. The top and bottom screenshots were taken from the simulations of rat and human vocal fold injury and repair, respectively. In each iteration, only a compressed image is sent over the network instead of sending the whole output data set. This approach allows a fast and efficient transfer of comprehensible outputs to the client. The image transfer costs are the same regardless of the simulation size. Clients only need to install a thin client package to see the visualized results. The 2D charts show total chemical aggregated statistics. Left most 3D volume in human simulation displays the distribution of one of the eight chemicals selected by the user. The second and third volumes show macrophage (brown) and neutrophil (red) distributions, respectively. The last volume on the right displays the tissue damage distribution (pink) and the distribution of fibroblasts (blue). Cell color codes are the same for both rat and human VF-ABM simulations.

A client-server *in situ* visualization protocol was employed to bypass the disk storage and provide users the ability to steer computation in real-time. For a seamless simulation and visualization experience, the latency of the server-client visualization pipeline had to be kept as minimal as possible even when a large amount of data is being simulated and visualized. One possible approach is to redirect OpenGL commands to the remote X server on the client side (Project, 2015). However, this approach puts significant loads on the network due to the transferring of both OpenGL calls and 3D data from the server to the remote client. Moreover, this approach strains the client with all of the rendering responsibilities, making the approach only suitable for applications with small and static data or specifically tuned OpenGL applications (Project, 2016). Another possible approach would be to use remote display software. However, some remote display software either lack the ability to run OpenGL applications, or force OpenGL applications to use a slow OpenGL software renderer (Project, 2016). Due to the size of the data produced by the 3D VF-ABM, the most suitable candidate is VirtualGL. The open source package, VirtualGL, allows any Unix or Linux remote display software to display OpenGL applications on the client's machine, while taking full advantage of the server's 3D graphics accelerators (Project, 2015). The OpenGL commands and 3D data are redirected to a 3D graphics accelerator on the server by VirtualGL. Thus, instead of sending a large amount of data points over the network, only one single simulation image frame (shown in Figure 4), which was visualized on the server, is sent to the client in each iteration. Given that this protocol shifts most of the rendering loads to the server, the client can take full advantage of the server's hardware, which is usually much more powerful than that of the client's machine. The employment of VirtualGL thus enhances the speed of the visualization through the server's accelerators without costing the client much hardware overhead.

## 3. Results

The simulation speed and accuracy are critical in making any biological model clinically useful. This section starts by examining the overall performance of the ABM simulation for our case study of the 3D VF-ABM, thereby illustrating the scalability of the model with respect to the number of cores available. The impact on the simulation accuracy with respect to the computational enhancement is then reported. Section 3.1.3 analyzes the performance of the 3D VF-ABM simulation suite and benchmarks its performance against existing ABM frameworks. Finally, the verification of model outputs is reported in section 3.2.

### 3.1. Performance evaluation

To optimize the overall simulation suite, each simulation component underwent aforesaid optimization techniques. Each technique was tailored to the specific computation and data access patterns of the respective component. Thus, their effects on performance were studied with respect to computation, visualization, and coupled simulation-visualization.

#### 3.1.1. Computational component

**Figure 5:**
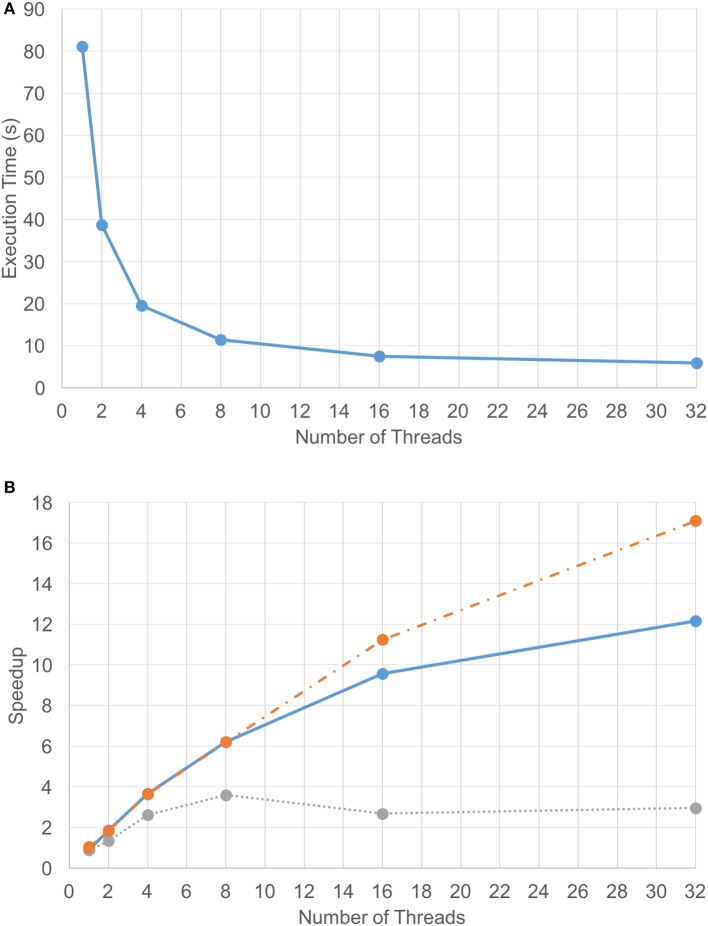
Computation-only performance scalability. Graphs showing **(A)** execution time and **(B)** speedup of the 3D VF-ABM for different number of threads. Notice that the average speedup of model function routines (orange-dotted) is much higher than the average speedup of the update routines (gray-dotted). The model function routines performed more computations than memory access operations, while the update routines performed more memory access operations than computations. As a result, a good scalability in model function routines was obtained but the scalability of update routines were relatively poor. Despite the memory-bound update functions, the overall speedup of the program (blue-solid) is still satisfactory.

Due to the efficiency of the FFT-based diffusion method, diffusing 1.2 billion point chemical data on two GPUs only took 2.5 s per iteration. However, the set of coarse-grain tasks (excluding updates) took about 4 s to execute. As a result, the coarse-grain tasks became the performance bottleneck. That is, the time that the VF-ABM takes to complete the computational component of a single iteration depends on how long it takes to execute the cellular tasks plus the time to synchronize the results. Figure 5A shows the execution time for the compute component using different numbers of CPU threads overlapping with two GPUs. These results indicate that the best performance using 32 threads takes approximately 6.2 s per iteration on average. The average speedup of the computational component as well as the speedups of its two main sub-components across 240 iterations over different numbers of threads are plotted in Figure 5B. Tasks were grouped into model functions (cell/ECM/synchronization) and update routines, and their speedups within each respective group were averaged. Notice that the update tasks consisted mostly of memory access operations. These operations were memory bound, thus showing poor scalability. Memory bound refers to the problem of memory speed not being able to keep up with the processor speed (McKee, 2004). The memory speed thus becomes the bottleneck of applications with low ratio of number of computation operations to number of memory operations. In contrast, other model function tasks involved more computation, and thus these tasks showed good scalability, making the overall speedup of the simulation reasonable.

#### 3.1.2. Visualization component

The coarse-grain tasks (excluding updates) took about 4.7 s to complete on the CPU. On the other hand, the fine-grain tasks on the GPUs only took 2.5 s. This difference in execution time resulted an idle period on the GPUs. If the visualization component was fast enough, this window would allow us to integrate visualization with the GPU computation without increasing the total execution time.

**Figure 6:**
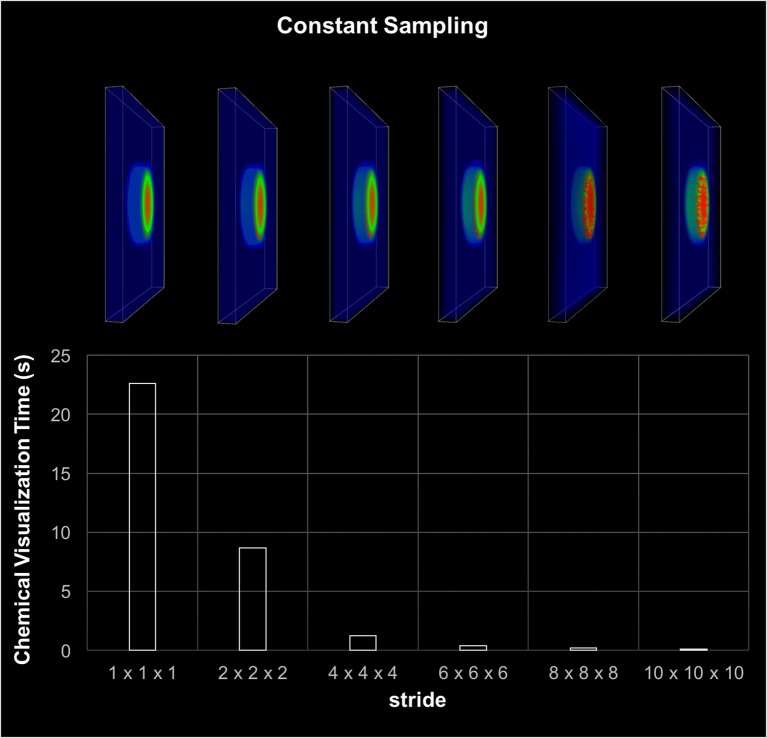
Visualization-only performance. This chart shows visualization screenshots and corresponding execution time for different sampling resolutions. The stride denotes the gap between two consecutive sampled points, thus the higher the stride the coarser the sampling. The visual appearance of the each sampling case looks almost identical for up to stride 6 or 6^3^ sampling windows. The visualization was able to retain sufficient information by using 6^3^ sampling.

The visualization component included the rendering of cell migration, chemical diffusion, and tissue damage tracking. The most time consuming component was the chemical diffusion, which required an access of 154 million points of data during each iteration. As discussed earlier, data sampling was used to improve the visualization performance. Figure 6 shows the execution time and screenshots of chemical visualization using different sampling window widths. The visualization of the entire world looked almost identical for up to 6^3^ sampling windows. Results showed that, looking at the entire simulation area, enough visual information was retained by using a fixed 6^3^ sampling window. However, if the user needed to zoom in to highly active areas, a more sophisticated adaptive sampling technique could be used instead of the fixed sampling used here (Seekhao et al., 2017).

#### 3.1.3. Coupled simulation and visualization

Since the visualization execution time was reduced from 23 s down to 0.4 s using data sampling for chemical diffusion, the visualization execution could then be placed in the idle period on one of the GPUs. By placing the visualization execution in a GPU idle gap, the total execution time remained unchanged at 6.2 s per iteration on average. This fast execution time enabled the simulation to execute remote computation, remote visualization, remote transmission of the result frame, and frame rendering on the client's machine in under 7 s/frame. This performance, as far as we know, is the fastest known complex ABM simulation and visualization at a similar scale.

**Figure 7:**
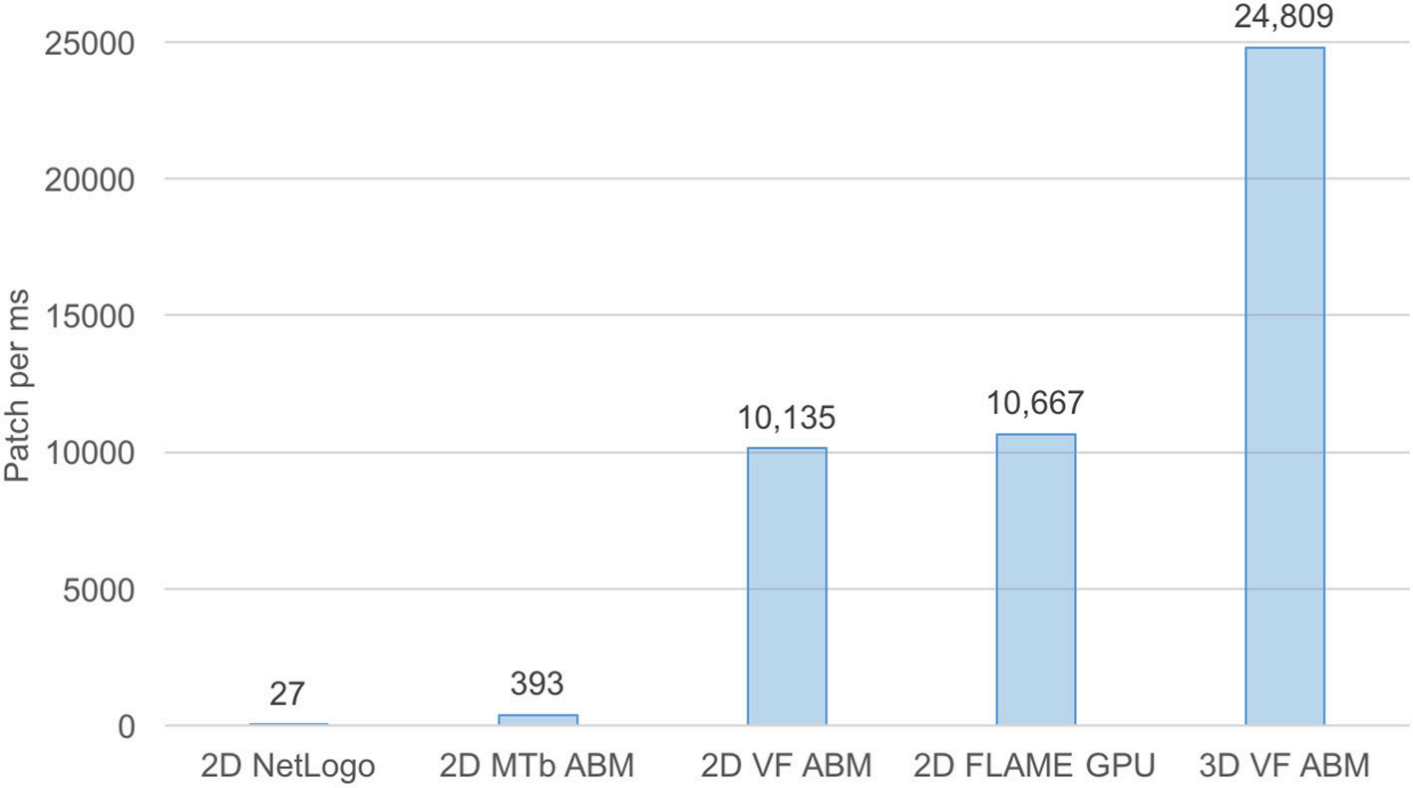
Processing power of 3D VF-ABM vs. existing work comparison. This bar chart compares workload and execution time in terms of number of patches (i.e., lattice points, grid points, stationary cells) per ms between the 3D VF-ABM to other bio-simulation ABM work. Notice that the 3D VF-ABM is capable of processing 25K patches/ms, or about 900x, 63x, 2.3x, and 2.4x more patch processing power than NetLogo, MTb ABM (D'Souza et al., 2009), FLAME GPU immune system ABM (de Paiva Oliveira and Richmond, 2016), and the earlier 2D VF-ABM work (Seekhao et al., 2016).

For benchmarking purposes, the 3D VF-ABM was compared to our previous and other ABM works of similar nature (Figure 7). The *M. Tuberculosis* (MTb) ABM (D'Souza et al., 2009) was benchmarked on a system with an NVIDIA GeForce 8800M GTX GPU, while GeForce GTX Titan was used for FLAME GPU immune system ABM (de Paiva Oliveira and Richmond, 2016). Despite the differences in underlying hardware, MTb ABM simulation is arguably one of the most suitable works for performance comparison with the 3D VF-ABM. The 2D MTb ABM simulated a complex multi-scale biological system of agents that communicate via chemical signals, which aligned in most respects with the 3D VF-ABM. The human immune system ABM was built on a widely used HPC ABM platform, FLAME GPU (de Paiva Oliveira and Richmond, 2016). Although this ABM executed the immune system at a much smaller timescale, the cell communication method is similar to other ABMs included in this performance comparison, i.e., communication via chemical signals. The FLAME GPU immune system ABM thus served as a good performance reference.

The 3D VF-ABM was simulated at a scale physiologically representative of a human vocal fold. Such scale was not feasible to be implemented on ABM freeware NetLogo (Wilensky and Evanston, 1999). Furthermore, to our best knowledge, no similar scale had been reported in any other publication. For a common throughput unit, the simulation performance was measured in terms of environment *space unit per millisecond*. The space units represent the smallest granularity of the ABM environment. Depending on the model, the space units can be patches (Wilensky, 2015), grid points (D'Souza et al., 2009), or immobile tissue cells (de Paiva Oliveira and Richmond, 2016). These quantities determine the ABM environment size. Therefore, the number of space units are proportional to the amount of work required to simulate the ABM environment in each iteration. For this reason, *space unit per millisecond* serves a reasonable throughput measure. The 3D VF-ABM is capable of processing 25K patches/ms, which is about 900x, 63x, 2.3x, and 2.4x the throughputs of NetLogo, MTb, FLAME GPU immune system ABM and the 2D VF-ABM, respectively. The comparison of the model scale, complexity and performances are in Table 5. Of note, FLAME GPU can process roughly 1.9x more mobile agents than 3D VF-ABM per time unit. The primary reason was that the time step used in FLAME GPU immune system ABM are smaller than that of our model in orders of magnitudes. This time scale difference caused their agent rules to be much less complex. For example, FLAME GPU immune system ABM would take roughly 18 h to complete a 5-day simulation while the 3D VF-ABM only takes less than half an hour. In addition, the 3D VF-ABM offered a much more rigorous data visualization in real-time at a scale of over 100 times more mobile agents than that of FLAME GPU immune system ABM.

**Table 5:** Performance and scale comparison with existing high-performance ABM work of similar nature.

	**# Patches**	**# Agents**	**# Chemical types**	**# ECM protein types**	**Time step**	**Average execution time per Iteration**
2D MTb ABM	16.4 K	3.2 K	1	0	10 min	0.042
2D NetLogo VF-ABM	1.0 M	114.0 K	8	3	30 min	36.6
2D FLAME GPU	320.0 K	160.1 K	1	0	0.2 s	0.03
2D VF-ABM	1.9 M	228.0 K	8	3	30 min	0.19
3D VF-ABM	153.8 M	16.9 M	8	3	30 min	6.2

### 3.2. Verification

The trends of the 3D VF-ABM output were *qualitatively* verified using the pattern-oriented analytical approach (Railsback, 2001; Grimm et al., 2005; Li et al., 2010b). The purpose of qualitative verification was to ensure that the dynamics of the model reflect what is expected in the wound healing literature and the available experimental data (Railsback, 2001; Grimm et al., 2005; Lim et al., 2006; Welham et al., 2008).

**Figure 8:**
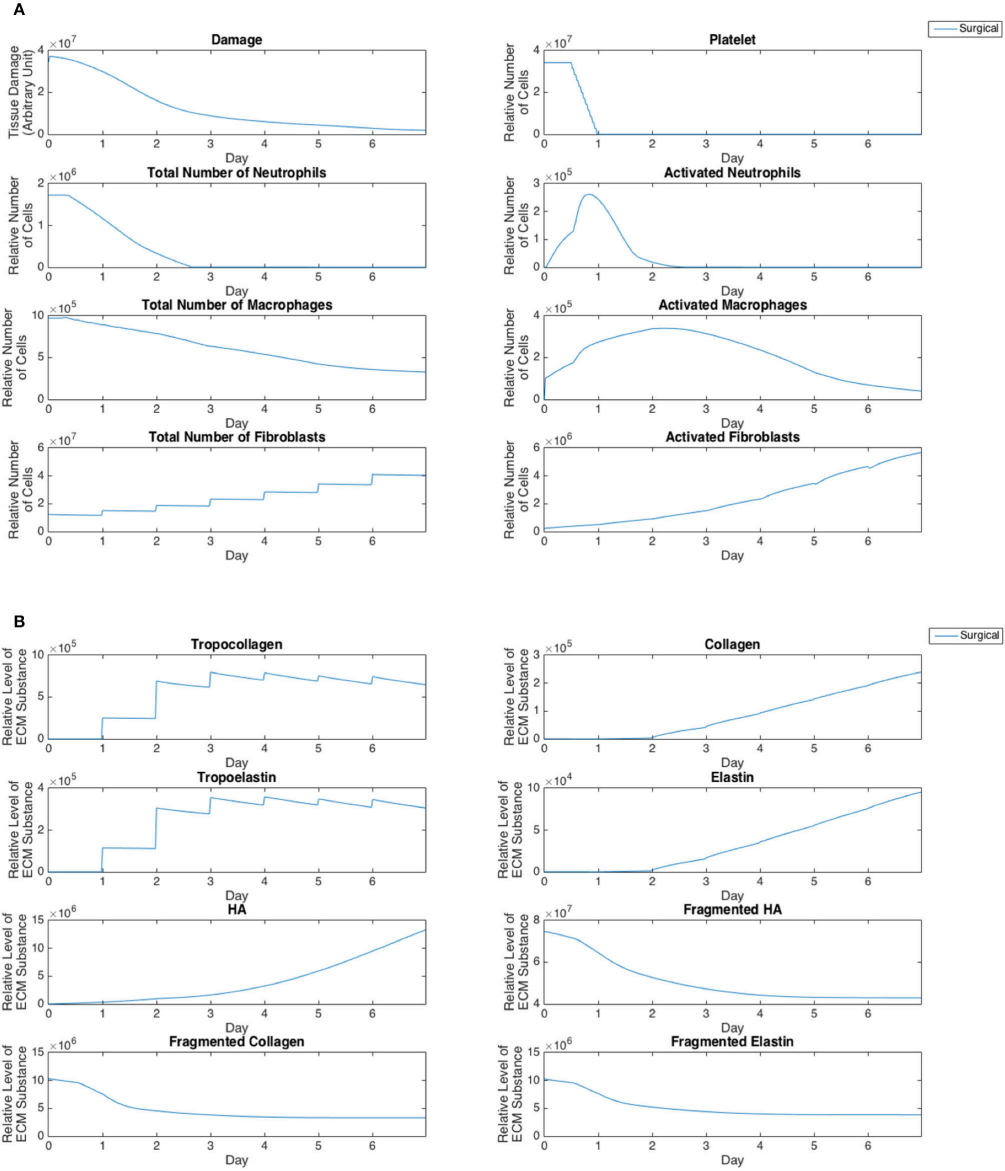
Simulation outputs. **(A)** Tissue damage and cell populations. **(B)** ECM subtances.

Cell population and ECM protein trends were compared against known patterns reported in wound healing literature as summarized in Table 6 (Martin, 1997; Witte and Barbul, 1997; Robson et al., 2001; Cockbill, 2002; Tateya et al., 2005; Dechert et al., 2006; Stern et al., 2006; Tateya I. et al., 2006; Tateya T. et al., 2006; Jiang et al., 2007). Figure 8 shows cellular and molecular outputs of the VF-ABM from a 7-day simulation. The model predicted a peak neutrophil population at the end of day 1 and significant decreases in day 2. The model also reproduced a peak of macrophage population around day 2 and a downward trend from the beginning of day 3 onward. Furthermore, the fibroblast proliferation started around the end of day 1 in the simulation. Trends of these specific cell populations agreed well with the known patterns in wound healing literature (Table 6). For ECM outputs, the VF-ABM reproduced the trends of collagen but not of hyaluronan. In particular, both empirical and ABM results showed the accumulation of collagen starting from Day 3. The ABM predicted an earlier accumulation of hyaluronan (Day 1) compared to empirical data (Day 3). This early hyaluronan accumulation might be related to high levels of TNF-α, TGF-β, FGF, and IL-1β that stimulated the secretion of hyaluronan by fibroblasts in the model. More data and calibration are needed for further investigation.

**Table 6:** Summary of patterns used for qualitatively verify 3D VF-ABM (Li et al., 2010b).

**Validation patterns**	**Source References**
Neutrophils arrive at wound site in first few hours	Martin, 1997; Witte and Barbul, 1997; Robson et al., 2001; Cockbill, 2002
Neutrophil number is at maximum by day 1 or 2	Martin, 1997; Witte and Barbul, 1997; Robson et al., 2001; Cockbill, 2002
Neutrophil number decreases rapidly around day 3 or 4	Martin, 1997; Witte and Barbul, 1997; Robson et al., 2001; Cockbill, 2002
Macrophage number is at maximum by days 2 to 4	Martin, 1997; Witte and Barbul, 1997; Robson et al., 2001; Cockbill, 2002
Fibroblasts start proliferation on day 1	Tateya I. et al., 2006
Fibroblast number decreases significantly on day 7 and stays low until day 14	Martin, 1997; Witte and Barbul, 1997; Robson et al., 2001; Cockbill, 2002; Tateya I. et al., 2006
Hyaluronan is first seen on day 3 and peaks at day 5 and starts to drop significantly at day 7, and then remains at low level until day 14	Tateya et al., 2005; Dechert et al., 2006; Tateya T. et al., 2006; Jiang et al., 2007
Peak of accumulated hyaluronan content occurs at the same time as peak of inflammatory cells (neutrophils and macrophages)	Stern et al., 2006; Jiang et al., 2007
Hyaluronan level is generally lower than for uninjured vocal folds after injury throughout healing period	Tateya et al., 2005; Tateya T. et al., 2006
Collagen type I curve is sigmoid-shaped	Witte and Barbul, 1997; Robson et al., 2001
Collagen type I is first seen on day 3 and peaks on day 5	Tateya et al., 2005; Tateya T. et al., 2006
Collagen type I level is generally higher than for uninjured vocal folds after injury throughout healing period	Tateya et al., 2005; Tateya T. et al., 2006

**Figure 9:**
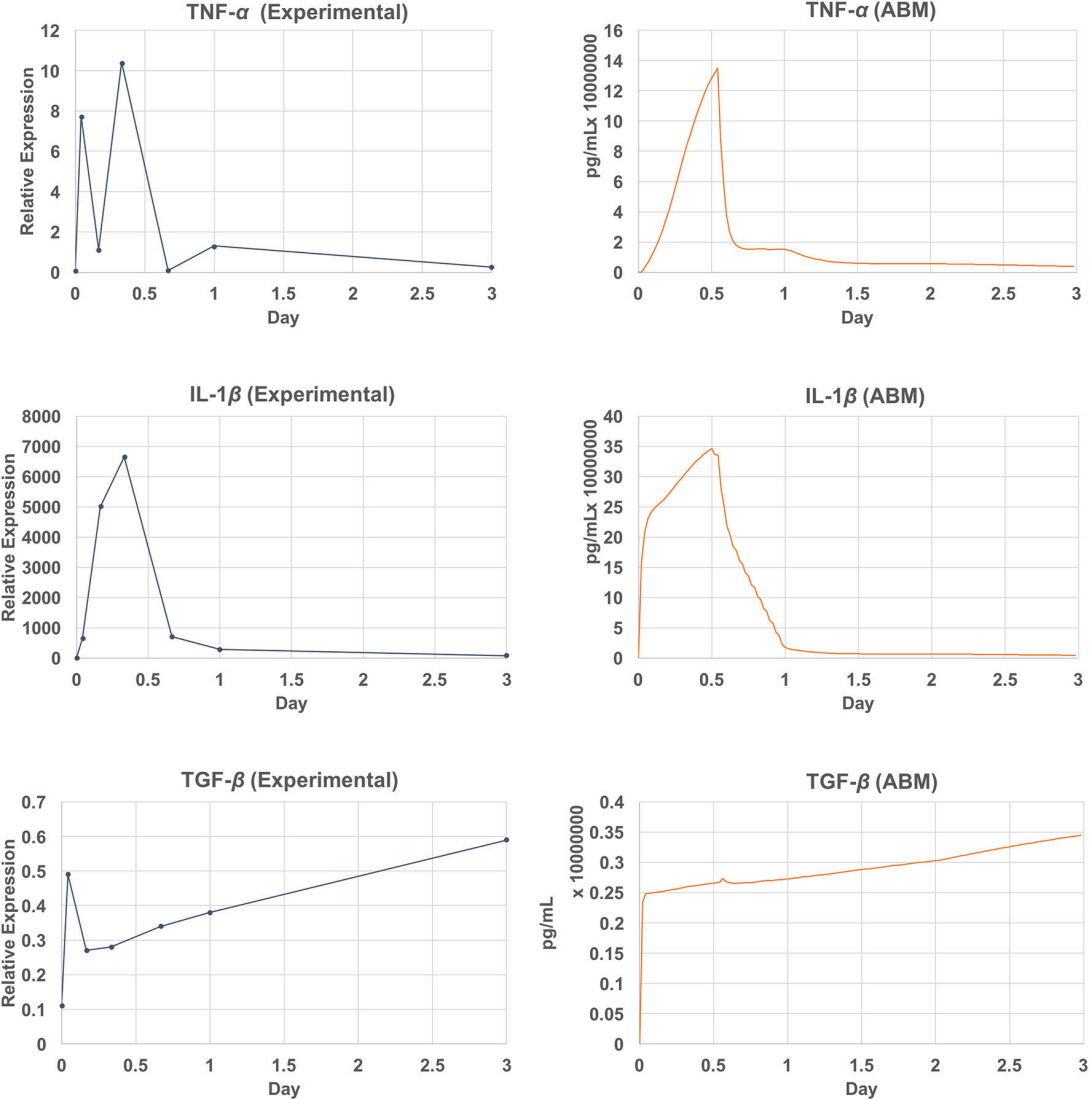
Empirical data vs. simulation output plot. Qualitative verification of the model output **(left)** against empirical data (Lim et al., 2006; Welham et al., 2008) **(right)**. The set of verified chemicals includes TNF-α, TGF-β, and IL-1β.

Due to the data availability, only a subset of chemicals was compared against the empirical data (Lim et al., 2006; Welham et al., 2008). This subset includes measured mRNA levels of three inflammatory mediators (TNF-α, TGF-β, and IL-1β) out of 8 that are simulated by the model. The comparison of the model outputs and the empirical data are shown in Figure 9. The ABM generated a peak of TNF-α after 13 h (26 ticks) of injury, whereas this peak occurred at hour 8 (tick 16) in the empirical data. For IL-1β, the model generated a peak at hour 12 (tick 24), where the peak was observed at hour 8 in the empirical data. Overall, the ABM-predicted peaks for TNF-α and IL-1β lagged behind the experimentally observed peaks by 4–5 h. The discrepancy between the model outputs and literature data may be explained as follows. First, since TNF-α and IL-1β were down-regulated by TGF-β and IL-10 via macrophages and fibroblasts, a possible reason for the peak delay could be an insufficient strength of TGF-β or IL-10. Second, since no empirical data were reported between hour 8 and 16, a peak between this interval might have been missed experimentally. More empirical data are needed for further investigation. For TGF, the model missed predicting the spike at hour 1. However, the sub-linear upward trend from hour 4 till the end of the simulation predicted by the model matched with that of the empirical data. In sum, the VF-ABM trajectories of inflammatory mediators showed a few discrepancies when comparing with the empirical vocal fold data in literature. Despite these few discrepancies, the overall dynamics of the VF-ABM outputs are consistent with those seen in the empirical data. Note that for this VF-ABM to be clinically ready, more experimental data is needed to calibrate the model. Future directions of this line of work will be discussed later in section 4.

## 4. Discussion

This work presents novel 3D ABM implementation techniques to tackle the heterogeneity of time scales in large-scale and multi-scale computational biology modeling. This 3D ABM for complex biological systems harnessed high-performance computing techniques to accommodate high-resolution models in simulating the model geometry and cellular components in the full physiological dimension without having to scale down the problem size. Kernel volume reduction was used to speed up convolution-based fine-grain chemical diffusion tasks on the GPUs. OpenMP was used to parallelize the coarse-grain cellular tasks the CPU cores. A task scheduling scheme was then used to overlap and synchronize the coarse-grain, fine-grain diffusion and *in situ* visualization components. This approach incurred optimal concurrent utilization of both multi-core CPUs and GPUs, resulting in minimal hardware resource idle time. The 3D VF-ABM prototype demonstrated tremendous performance improvements to high-resolution cellular-level models achieved with the proposed scheme. The high-performance simulation suite is capable of large-scale computing and remote visualization in under an average of 7 s per iteration. The computational component tracks 17 million cells and process 1.7 billion signaling chemical and structural protein data points. The remote visualization component renders 17 million cells and 154 million signaling chemical data points on the server then send result frame to the user. Compared to related work of similar nature, the 3D VF-ABM showed roughly 900x, 63x, and 23x data processing power over the NetLogo version of vocal fold ABM, MTb ABM (D'Souza et al., 2009), and FLAME GPU immune system ABM (de Paiva Oliveira and Richmond, 2016), respectively.

Model verification of the VF prototype was perform *qualitatively* against known patterns (Martin, 1997; Witte and Barbul, 1997; Robson et al., 2001; Cockbill, 2002; Tateya et al., 2005; Dechert et al., 2006; Stern et al., 2006; Tateya I. et al., 2006; Tateya T. et al., 2006; Jiang et al., 2007), and against rat vocal fold surgical data (Lim et al., 2006; Welham et al., 2008). The model reproduced the overall dynamics of cellular and molecular trajectories seen in surgical vocal fold injuries. However, in a few cases, such as the trends of hyaluronan and collagen, the model missed predicting their peaks. This mismatch between the model and empirical trends was possibly caused by imbalances in the levels of regulating substances. More data and further calibration process are required to investigate this matter.

As discussed earlier, our ABM world currently only supports regular grids and thus FDM applies well to the diffusion computation. An arbitrary shape world is a possible direction of future work that is yet to be explored. A technique such as indirect addressing (Randles et al., 2015) and advanced data structures such as octrees or meshes are examples of possible approaches to an ABM world geometry solution. These techniques clearly offer a more realistic representation of the real-world geometries but will also increase the model complexity. Simple FDM for diffusion may not apply well to these complex geometries. Variations of FDM (Hunt, 1978; Liszka and Orkisz, 1980) and other PDE approximation schemes such as finite element method (FEM) should thus be considered in future ABM developments.

Ongoing work on parallelizable calibration automation is being developed to refine the parameter values of the VF-ABM with additional vocal fold data collected in our laboratory (Li et al., 2012; Heris et al., 2015; Latifi et al., 2016; Li-Jessen et al., 2017) and others (King et al., 2015; Kishimoto et al., 2016). Those works are necessary to improve the biological representation of the VF-ABM for the ultimate clinical application. High-performance techniques are being expanded to facilitate more complex data explorations such as active area resolution enhancement (Seekhao et al., 2017), 3D volume rendering of ECM protein content, tissue fiber orientation and structure, while still maintaining real-time performance. This work focuses on the application of surgical vocal fold injury and repair because the empirical data (Lim et al., 2006; Welham et al., 2008) are available for model verification. However, the host-accelerators (CPU-GPUs) coordination, diffusion kernel reduction, and other techniques proposed here can be generalized and applied to other complex multi-scale biological system applications to enhance their performance on heterogeneous HPC platforms.

## Author contributions

NS and JJ: conceived of the presented ABM optimization ideas; NL-J: designed the VF-ABM rules and the overall concept of the project; CS: designed and implemented the sequential version of the 3D VF-ABM; NS: designed, implemented, and benchmarked the parallel version of the 3D VF-ABM; NS: designed and developed the visualization component of the 3D VF-ABM; NS: analyzed model-generate outputs and performed the qualitative verification; JJ, NL-J, and LM: supervised the project; NS: wrote the manuscript; JJ and NL-J: revised the manuscript critically; JJ, NL-J, and LM: provided funding and computing resources to support the project. All authors provided critical feedback on the project and the manuscript.

### Conflict of interest statement

The authors declare that the research was conducted in the absence of any commercial or financial relationships that could be construed as a potential conflict of interest.
